# Open Lumbar Spine Fracture-Dislocation in a Rural Setting Associated With a Fall From Height: A Case Report

**DOI:** 10.7759/cureus.116272

**Published:** 2026-09-15

**Authors:** Luis G Assis

**Affiliations:** 1 Orthopaedics and Traumatology, Universidade Federal da Fronteira Sul, Passo Fundo, BRA

**Keywords:** case report, fracture-dislocation, lumbar fractures, lumbar fusion, spinal cord injury, spinal fusion

## Abstract

Open spinal fractures are rare and highly complex injuries associated with a high risk of infection and neurological impairment. We report a case of a previously healthy 25-year-old male who sustained a fall from approximately four meters in a rural setting, with direct impact of the lumbar region against a toilet, resulting in an extensive wound contaminated with feces, urine, soil, and ceramic fragments. Upon admission, he presented with an approximately 15-cm lacerated and contused wound, disruption of the paravertebral fascia, and exposure of the posterior spinal elements. Computed tomography revealed comminuted fractures of the posterior elements of L3, L4, and L5, with bone fragments displaced into the spinal canal and approximately 50% segmental stenosis, associated with injury to the posterior ligamentous complex. Neurological examination was normal, classified as Frankel grade E/American Spinal Injury Association (ASIA) grade E, with a Thoracolumbar Injury Classification and Severity Score (TLICS) of 7 points. Extensive surgical debridement, irrigation with more than 10 liters of saline solution, foreign-body removal, spinal canal decompression, repair of an approximately 2-cm dural tear, and posterior stabilization from L2 to S1 with pedicle screw instrumentation were performed. Due to the extensive soft-tissue injury, negative-pressure wound therapy was used. Wound cultures identified *Proteus mirabilis*, and targeted antibiotic therapy was administered for 28 days. The patient had no deep infection or neurological deficit and demonstrated satisfactory wound healing, bony fusion, and functional recovery at 180 days of follow-up. This case highlights the importance of early debridement, copious irrigation, contamination control, targeted antibiotic therapy, and surgical stabilization in open lumbar spinal fractures.

## Introduction

Open spinal fractures are rare injuries, generally associated with high-energy trauma, and are characterized by instability, extensive soft-tissue injury, and a high risk of infection. Reports of open lumbosacral fracture-dislocations demonstrate the complexity of treatment, particularly in the presence of contamination and neural structure injury [1].

Computed tomography allows characterization of fracture morphology and assessment of posterior ligamentous complex involvement. The Thoracolumbar Injury Classification and Severity Score (TLICS) assists in evaluating spinal stability and guiding treatment, whereas the AO Spine classification contributes to the description of the injury [1-5].

We report a case of an open lumbar fracture-dislocation following a fall from height in a rural setting, with extensive contamination by organic material and ceramic fragments, associated with a dural tear, and treated surgically.

## Case presentation

A 25-year-old previously healthy male sustained a fall from approximately four meters in a rural setting, with direct impact of the lumbar region against a toilet. The wound was exposed to feces, urine, soil, and ceramic fragments. He was referred to the emergency department four hours after the trauma.

On admission, he presented with an approximately 15-cm transverse lacerated and contused wound in the lumbar region (Figure 1), with loss of skin and subcutaneous tissue, disruption of the paravertebral fascia, and exposure of the posterior bony elements, with gross contamination. He was hemodynamically stable and had no motor or sensory deficits, corresponding to Frankel grade E/American Spinal Injury Association (ASIA) grade E [5,6].

**Figure 1:**
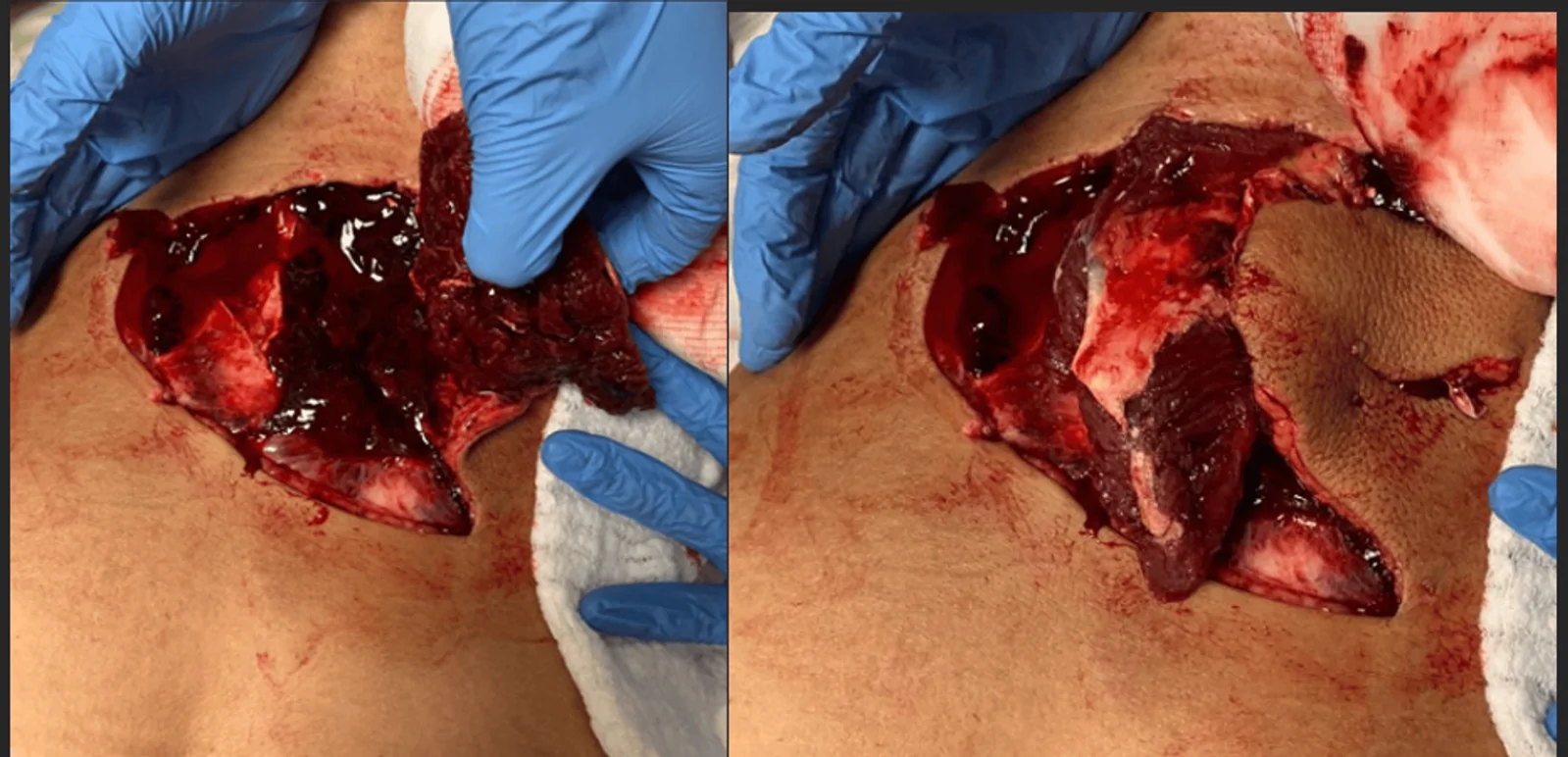
Lumbar wound at admission demonstrating exposure of the posterior elements with contamination by organic material and ceramic fragments.

Computed tomography demonstrated comminuted and displaced fractures of the spinous processes and laminae of L3, L4, and L5, with bone fragments displaced into the spinal canal, resulting in approximately 50% segmental stenosis, associated with facet injury and disruption of the posterior ligamentous complex (Figure 2). The TLICS was 7 points, indicating spinal instability and the need for surgical treatment [2-4].

**Figure 2:**
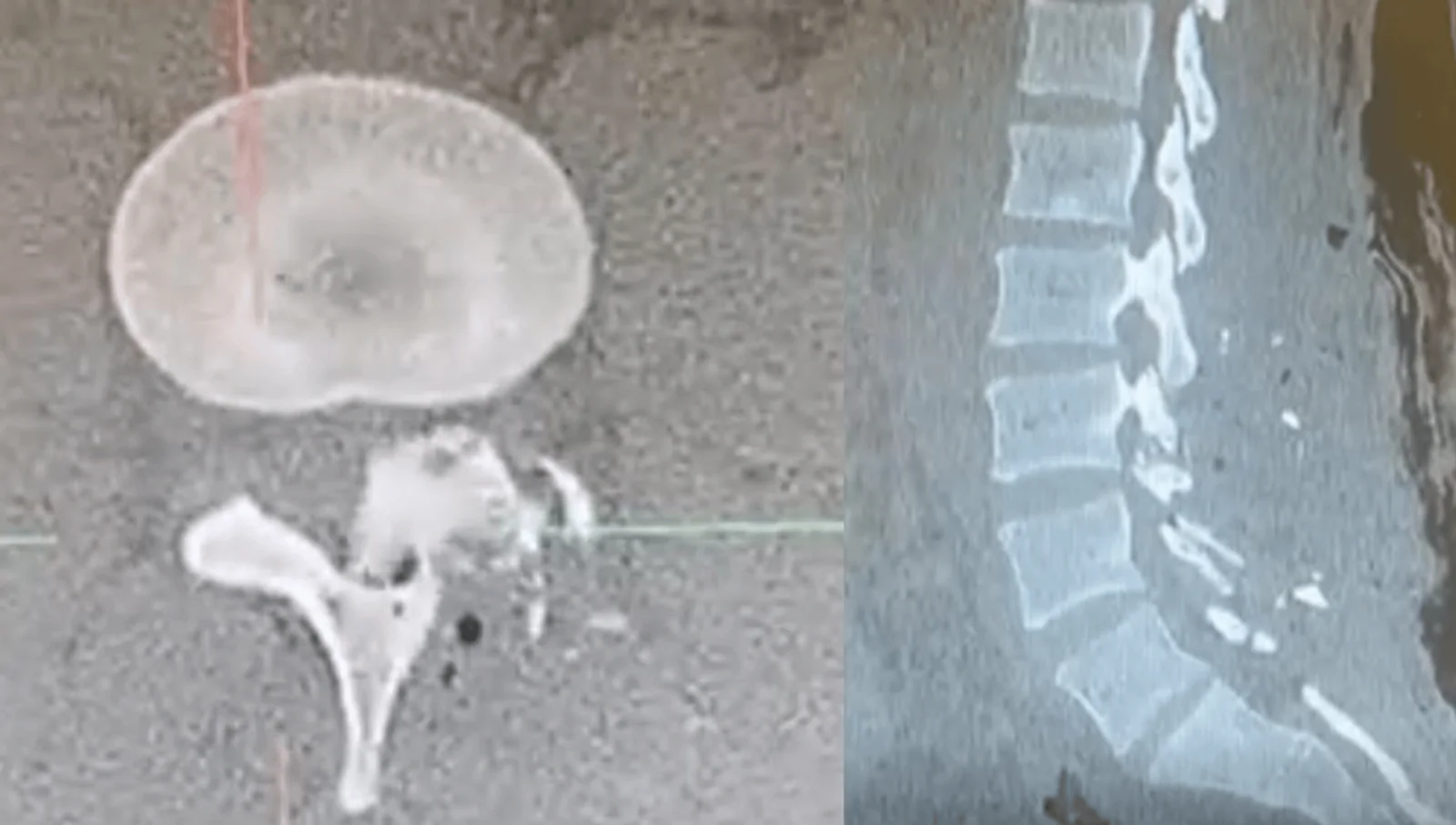
Computed tomography demonstrating fractures of the posterior elements of L3–L5, bone fragments within the spinal canal, and segmental stenosis.

Broad-spectrum intravenous antibiotic therapy was initiated, and urgent surgical management was performed. With the patient in the prone position, extensive debridement, foreign-body removal, and irrigation with more than 10 liters of 0.9% saline solution were performed. Intraoperative exploration revealed disruption of the paravertebral fascia, injury to the interspinous ligaments, and disruption of the left facet joint capsule at L4-L5 (Figure 3), in addition to extensive contamination with ceramic fragments, soil, and fecal material.

**Figure 3:**
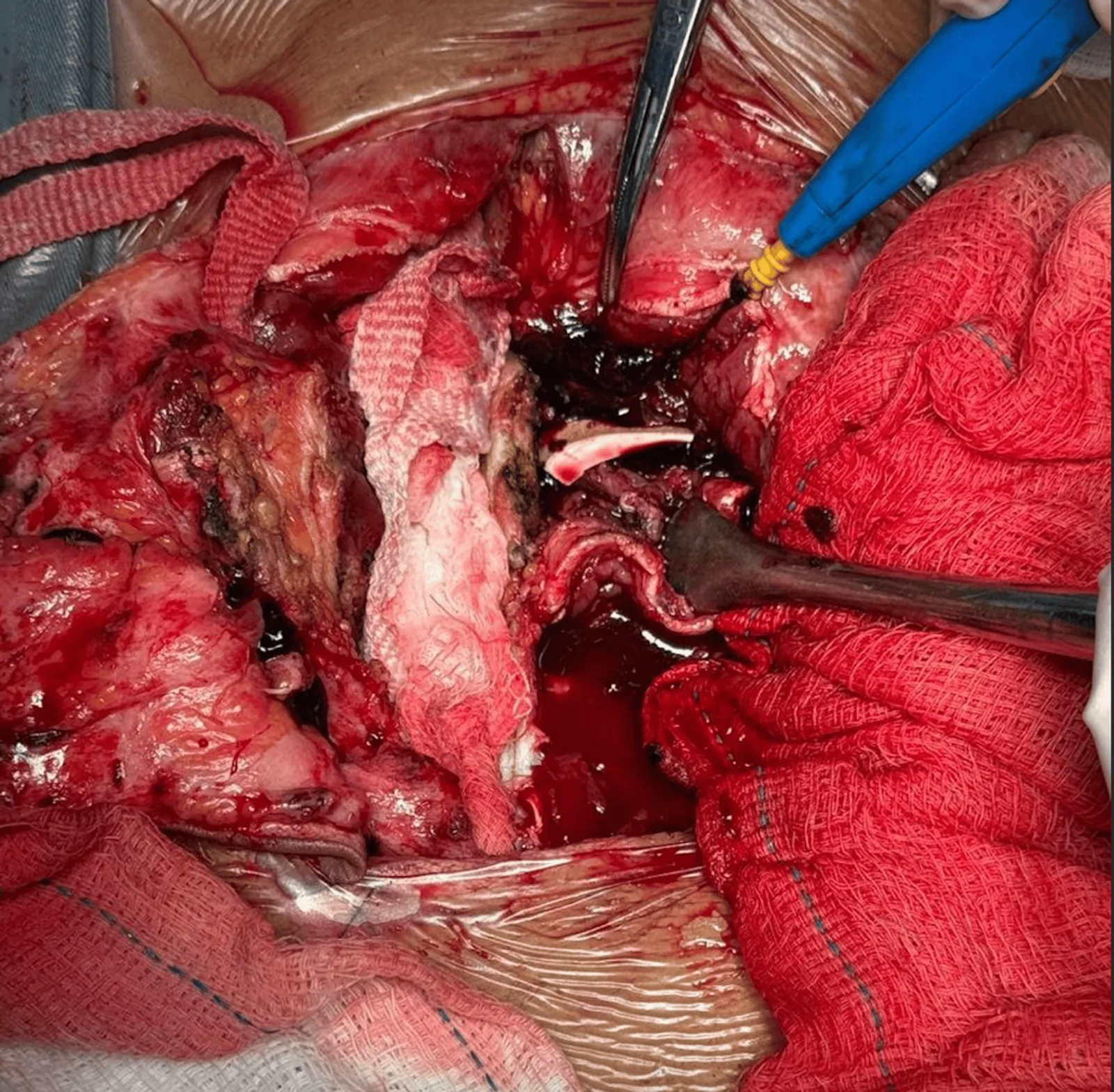
Intraoperative view showing fascial disruption and contamination with ceramic fragments and organic material.

A dural tear measuring approximately 2 cm was identified and repaired with sutures and dural sealant. Spinal canal decompression and reduction of the injury were performed, followed by posterior stabilization from L2 to S1 using pedicle screw instrumentation (Figure 4).

**Figure 4:**
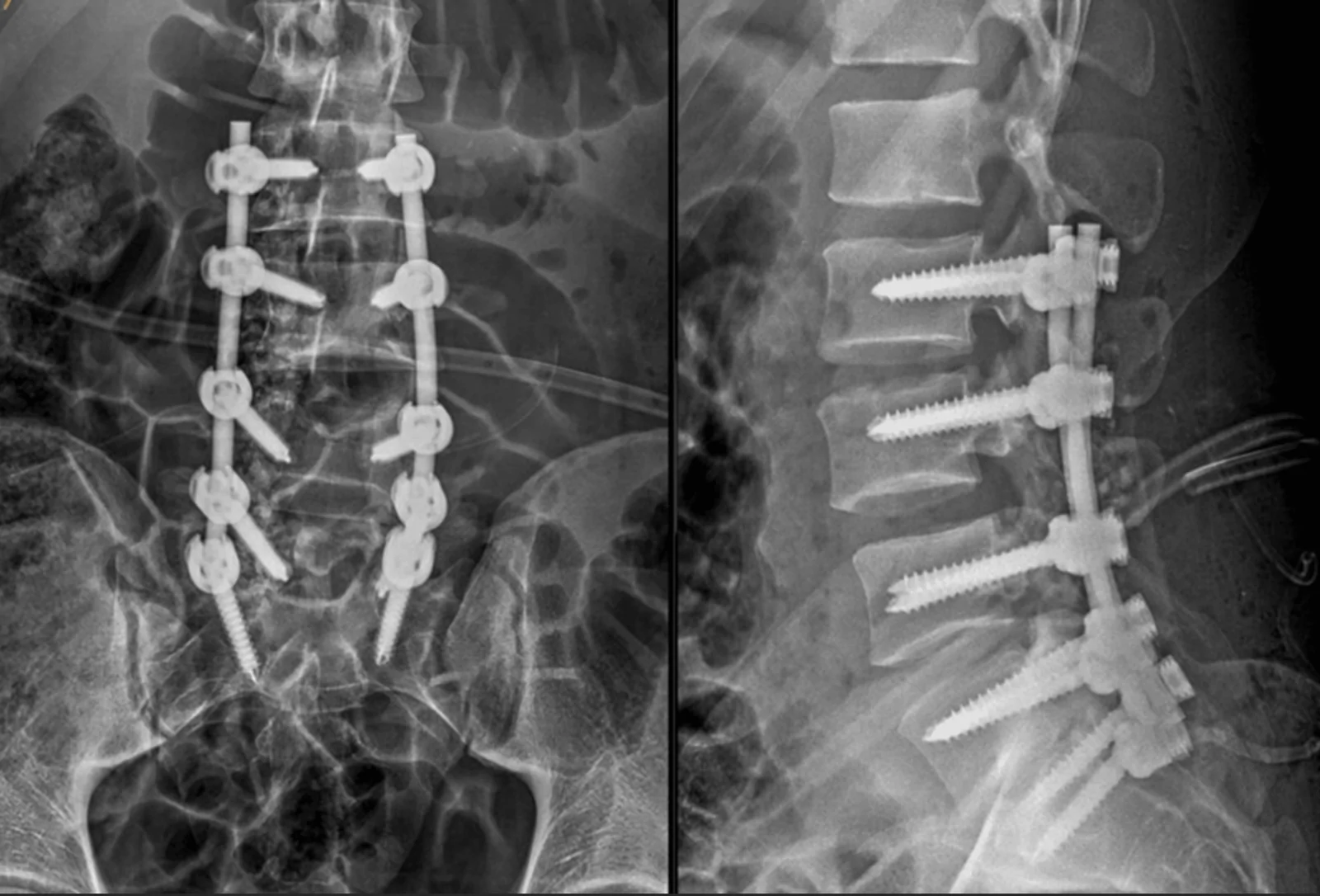
Postoperative radiographs demonstrating posterior stabilization from L2 to S1.

Due to the extensive soft-tissue injury, negative-pressure wound therapy was applied in association with a subfascial drain. The patient remained in the intensive care unit for five days and underwent early physical therapy. Wound cultures identified *Proteus mirabilis*, and antimicrobial therapy was adjusted according to the recommendations of the infectious disease team, with a total treatment duration of 28 days.

The patient had an uneventful postoperative course, without deep infection, cerebrospinal fluid leakage, or neurological deterioration. At discharge, motor strength and sensory function remained intact, with a Frankel grade E/ASIA grade E classification [5,6]. At 180 days of follow-up, he demonstrated good functional recovery, satisfactory bony fusion, and no infectious or neurological complications (Figure 5).

**Figure 5:**
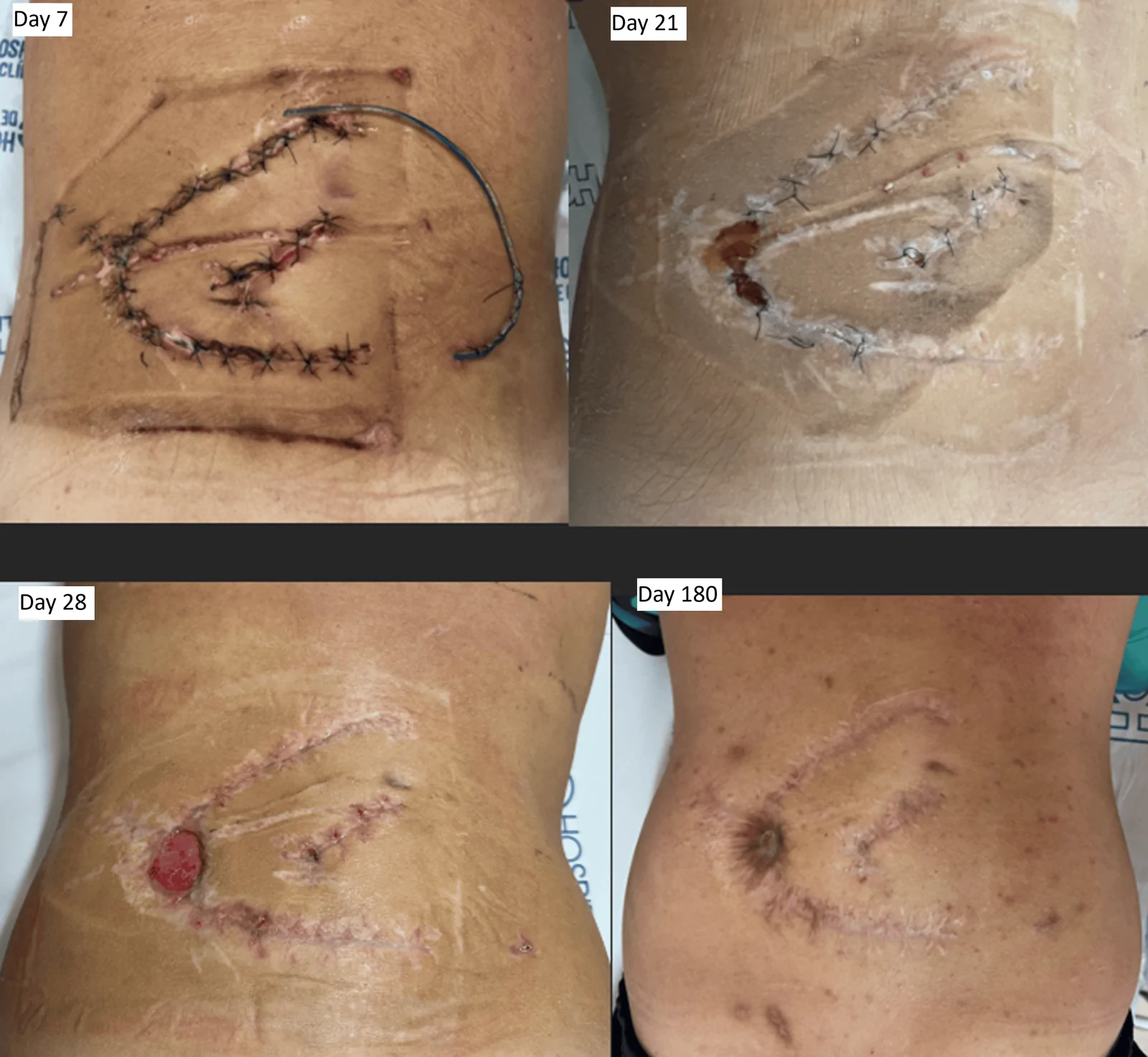
Wound healing progression on postoperative days 7, 21, 28, and 180.

 The clinical timeline of the patient is presented in Table 1.

**Table 1:** Clinical timeline. ASIA: American Spinal Injury Association; TLICS: Thoracolumbar Injury Classification and Severity Score.

Time point	Clinical event
Admission (4 hours post-trauma)	15-cm lacerated and contused lumbar wound, fascial disruption, exposure of posterior elements; Frankel grade E/ASIA grade E [5,6]; TLICS 7 [2-4].
Day 0 (surgery)	Extensive debridement, >10 L saline irrigation, foreign-body removal, dural tear repair (~2 cm), canal decompression, posterior stabilization L2–S1
Postoperative	Negative-pressure wound therapy applied with subfascial drain; ICU stay of 5 days; early physical therapy initiated
Culture result	*Proteus mirabilis* identified; targeted antibiotic therapy adjusted per infectious disease recommendations
Antibiotic course	Total duration 28 days
Discharge	Motor and sensory function intact (Frankel grade E/ASIA grade E); no deep infection or CSF leak
Day 7, 21, 28	Progressive wound healing documented
Day 180 (final follow-up)	Satisfactory bony fusion, good functional recovery, no infectious or neurological complications

## Discussion

Open lumbar spinal fractures are highly complex injuries due to the combination of instability, extensive soft-tissue destruction, and communication with the external environment. The specific literature on these injuries is limited and consists primarily of case reports and small case series [1].

In this case, contamination with fecal material, urine, soil, and ceramic fragments, combined with fascial disruption and a dural tear, represented a substantial risk of infection. Management was based on the principles of open fracture treatment, including early antibiotic therapy, debridement of devitalized tissue, copious irrigation, and adequate stabilization [7,8].

A TLICS of 7 points and disruption of the posterior ligamentous complex supported the indication for surgical stabilization [2-4].

Despite preserved neurological function, the presence of bone fragments within the spinal canal and spinal instability warranted decompression and stabilization.

Negative-pressure wound therapy was used as an adjunctive measure to manage the extensive soft-tissue injury. Antibiotic therapy was individualized according to the degree of contamination and microbiological results, as there is no universal consensus regarding its duration in contaminated spinal trauma [9,10].

## Conclusions

The open lumbar fracture-dislocation described in this case represents a rare and severe injury. Treatment with extensive debridement, copious irrigation, dural repair, targeted antibiotic therapy, posterior stabilization from L2 to S1, and negative-pressure wound therapy resulted in a satisfactory clinical course, with no deep infection or neurological deterioration during 180 days of follow-up.
